# Patients Refusing Prehospital Transport Are Increasingly Likely to Be Geriatric

**DOI:** 10.1155/2012/905976

**Published:** 2012-02-19

**Authors:** Peyton Holder, Annette O. Arthur, Grady Thiems, Travis Redmon, Matt Thomas, Jeffrey M. Goodloe, T. J. Reginald, Stephen H. Thomas

**Affiliations:** Department of Emergency Medicine, OU Schusterman Center, University of Oklahoma School of Community Medicine, 4502 East 41st Street Suite 2E14, Tulsa, OK 74135, USA

## Abstract

*Objective*. Elderly patients are becoming an increasingly larger proportion of our population, and there is a paucity of data regarding the epidemiology of geriatric patients refusing transport. Treatment refusal rates range from 5% to 15% in many studies. This study sought to test the hypothesis that geriatric patients constituted an increasing proportion of those persons refusing prehospital transport. *Methods*. This study was a retrospective analysis of data from a query of a large urban EMS service. *Results*. There were a total of 22,347 adult transport refusals recorded during the 16-month study period. Multivariate logistic regression incorporating covariates for sex, race, season, chief complaint, metropolitan region, and whether any treatment occurred prior to transport refusal confirmed the increasing likelihood of Period 2 patients being geriatric, as compared with Period 1 (OR 1.24, 95% CI 1.14–1.35, Wald *P* < .001). *Conclusion*. This data shows that despite controlling for these covariates, patients refusing transport in the second period of this study were nearly 25% more likely to be geriatric as compared to those in the initial 8 months of the study.

## 1. Introduction

Emergency medical services (EMSs) are the system that is responsible for the prehospital treatment and transportation. When EMS is activated, they appropriately respond by arriving to give on the scene care. Of course, patients then have the right to receiving treatment and transportation or refuse one or both, against the advice of the treating paramedic (AMA). Treatment refusal rates range from 5% and 15%, in many studies [1–3]. Refusal of care or transport may happen for many reasons such as the patient not feeling they need further care and financial restraints. There may be negative outcomes associated with refusing treatment/transport, such as a subsequent Emergency Department (ED) visit, hospital admission, or even death.

Despite an increased emphasis on geriatric care, the health status of this age range is still meager. Elderly patients are becoming an increasingly larger proportion of our population, and there is a paucity of data regarding the epidemiology of geriatric patients refusing transport. This would be an alarming trend if found and would then require further study to look at the etiology of the refusal. This study sought to test the hypothesis that geriatric patients constituted an increasing proportion of those persons refusing prehospital transport.

## 2. Methods

This study was a retrospective analysis of data from a query of a large urban EMS service associated with the Oklahoma City, OK, and Tulsa, OK, metropolitan areas. The study includes all EMS interactions from January 1, 2010, to May 31, 2011, with patients who were at least 18 years old. There were no exclusions for gender, race, or diagnoses. The data includes EMS vehicle region (Eastern or Western), month and year of EMS interaction, response outcome (treated at the scene, patient refused care, care transferred), chief complaint, gender, age in years, and race. No patient identifiers such as encounter number, medical record number, or name were included.

All adult transport refusals over 16 months were included, with the study timeline of 16 months divided into the first 8 months (Period 1) and the second 8 months (Period 2). Periods 1 and 2 patients were assessed with univariate categorical analysis for proportions with chi-square testing. Next, multivariate logistic regression was used to determine association between the study period and geriatric status (age 65 or older), after adjustment for covariates such as sex, chief complaint, metropolitan region (of the two areas served by the study EMS service), race, season, and whether any treatment occurred prior to transport refusal. Analyses were conducted with STATA 11 MP (StataCorp, College Station, TX); *P* was set at 0.05 for all analyses and 95% confidence intervals (CIs) were calculated for odds ratios (ORs).

## 3. Results

There were a total of 22,347 adult transport refusals recorded during the 16-month study period. Total refusals of 10,622 (48.0%) versus 11,725 (52.0%) occurred during Period 1 and Period 2, respectively. Of the total transport refusals 4,723 (21.1%) were ≥65 years old (2,104 versus 2,619 during Period 1 and Period 2, resp.). Patient characteristics of transport refusals are presented in Table 1. Univariate categorical analysis revealed that Periods 1 and 2 differed (*P* < .001) with respect to proportions of geriatric patients (19.8% versus 22.3% in Period 1 and Period 2, resp.). Multivariate logistic regression incorporating covariates for sex, race, season, chief complaint, metropolitan region, and whether any treatment occurred prior to transport refusal confirmed the increasing likelihood of Period 2 patients being geriatric, as compared with Period 1 (OR 1.24, 95% CI 1.14–1.35, Wald *P* < .001).  Table 2 provides a breakdown of all EMS responses by chief complaint, with trauma being the most common. The number and percentage of patients refusing transport within each individual age group is found in Table 3. The finding of increased likelihood of geriatric status in those patients refusing transport was also present when the 16-month dataset was analyzed for overall trend during the 16 individual months of the study (OR 1.02, 95% CI 1.01–1.03, *P* < .001).

Of the 22,347 transport refusals, no treatment was given before transport refusal in 19,552 instances (9,218 versus 10,334 in Period 1 and Period 2, resp.). Treatment was given before refusal in the remaining 2,795 instances. There was no significance found for the presence of treatment on transport refusal rates (OR 1.02, 95% CI 92–1.13).

## 4. Discussion

Prior anecdotal evidence suggests an increasing prevalence of geriatric patients refusing EMS transport [1–3]. The data from this study strengthens the validity of this claim. However, the etiology and impact of this trend remains unclear. Patient sex, race, seasonal variations, and chief complaint all offer a plausible potential impact on transport refusal rates. Yet, this data shows that despite controlling for these covariates, patients refusing transport in the second period of this study were nearly 25% more likely to be geriatric as compared to those in the initial 8 months of the study. Although comparing subsequent individual months has obvious limitations, this trend remained true when dividing and analyzing the total 16-month study period into 16 individual time periods (2% increase in geriatric likelihood over each month during the study period). This rate of increase is rather alarming and warrants further investigation. One possible explanation for this observed increase could be financial constraints. The continued rise in cost of ambulance transport and hospital care combined with fixed income levels of many geriatric patients may be serving as a barrier to patient willingness to be transported. This study did not account for the financial means of those individuals who refused transport. A follow-up study seeking to elicit the cause of this increased refusal rate among the geriatric population would be both interesting and potentially impactful on the development of new strategies to improve appropriate EMS utilization by the elderly. In conclusion, the data from this study demonstrated a nearly 25% increased likelihood of geriatric patients to refuse EMS transport from the first 8-month period to the second 8-month period when controlling for covariates. Further investigation into the characterization and impact of this trend is warranted.

## Figures and Tables

**Table 1:** Characteristics of patients refusing transport.

	Period 1	Period 2	Total
Number of refusals	10622	11725	22347
Male	4832	5227	10059
Female	5555	6293	11848
Race			
Asian/PI	105	115	220
Black	2106	2249	4355
Native Amer	201	232	433
Other	143	108	251
White	6909	7971	14880
Age ≥65 yo	2,104	2619	4723
Treatment prior to transport refusal	1404	1391	2795

**Table 2:** Breakdown of EMS responses by chief complaint.

CC	Freq.	Percent	Cum.
Abdominal Pain (Medical)	284	1.95	1.95
Allergic Reaction	105	0.72	2.67
Altered Mental Status	329	2.26	4.93
Animal Bites/Stings	138	0.95	5.88
Assault	779	5.35	11.23
Back-Pain (Medical)	68	0.47	11.70
Bleeding (Medical)	128	0.88	12.58
Burn	83	0.57	13.15
Cardiac	156	1.07	14.22
Cardiac Arrest	4	0.03	14.25
Catheter Complications	5	0.03	14.28
Chest Pain (ACS)	272	1.87	16.15
Chest Pain (Non-Cardiac)	306	2.10	18.25
Choking	296	2.03	20.28
Diabetic Emergency	1,108	7.61	27.90
Dizziness	249	1.71	29.61
Electrocution/Lightning	19	0.13	29.74
Environmental Cold/Heat	140	0.96	30.70
Eye Problems	51	0.35	31.05
Hazardous Material Exposure	57	0.39	31.44
Headache	174	1.20	32.63
Mental health/Psychiatric Illness	669	4.60	37.23
Near Drowning	3	0.02	37.25
OB-GYN Problems	37	0.25	37.51
Other…	952	6.54	44.04
Pain	739	5.08	49.12
Poisoning/Overdose/Ingestion	416	2.86	51.98
Pregnancy/Childbirth	22	0.15	52.13
Respiratory Arrest	2	0.01	52.14
Respiratory Distress	883	6.07	58.21
Seizures/Convulsions	737	5.06	63.27
Sick Person	1,114	7.65	70.92
Stroke/CVA/TIA	54	0.37	71.29
Syncope/Near Syncope	960	6.59	77.89
Trauma-Abdominal	33	0.23	78.12
Trauma-Altered Mental Status	24	0.16	78.28
Trauma-Breathing Problems	5	0.03	78.31
Trauma-Chest	75	0.52	78.83
Trauma-Multisystem	29	0.20	79.03
Trauma-Penetrating	83	0.57	79.60
Trauma-Other	2,494	17.13	96.73
Unconscious	12	0.08	96.81
Unresponsive	21	0.14	96.96
Weakness	443	3.04	100.00

Total	14,558	100.00	

**Table 3:** Refusal rate among individual age groups.

Age Range	Freq.	Percent	Cum.
0–11 Mos	2	0.01	0.01
1–4 yrs	613	4.32	4.33
5–10 yrs	493	3.47	7.80
11–16 yrs	693	4.88	12.68
17–21 yrs	1,340	9.44	22.12
22–30 yrs	1,908	13.44	35.56
31–40 yrs	1,867	13.15	48.71
41–50 yrs	1,951	13.74	62.45
51–60 yrs	1,784	12.57	75.03
61–70 yrs	1,287	9.07	84.10
71–80 yrs	1,054	7.43	91.53
81–90 yrs	902	6.35	97.88
91+ yrs	301	2.12	100.00

Total	14,195	100.00	

## Appendix

See Tables 1, 2, and 3.
